# The Making of a Modern Hospital


**Published:** 1912-03-16

**Authors:** 


### THE MAKING OF A MODERN HOSPITAL.*

### The Ward Windows and Doors.

The importance of adequate diffuse sunlight in the ward can scarcely be overestimated, and modern hospital architects are fully alive to the necessity of providing for such sunlight distribution throughout the ward by means of adequate window-space. Every modern hospital is so built that its long axis lies approximately south-east-north-west, so that its front and back walls are respectively facing south-west and north-east. By this disposition the largest amount of sunlight is secured in this country and generally in Europe. The importance of direct sunlight is due to the fact that sunlight is one of the most powerful disinfectants and deodorisers that is known. Even diffuse sunlight exerts this disinfecting power, while direct sunlight actively oxidises organic material and so secures the destruction of bacteria. That being the case, it has become an axiom with modern hospital constructors that " a modern hospital ward ought to be so planned that every square inch of floorage is directly sunlit at some period during the day." The hospital ward that does not conform to this rule can scarcely be regarded as up-to-date and modern in the best sense of the terms.

### Surface Lighting.

Such direct lighting is secured by laterally placed windows. The old practice of providing roof windows is now no longer followed; it is quite unsuitable for a hospital, however excellent it may be for a picture gallery. We have already in a former article dealt very briefly with the surface lighting of some typical hospitals, and there is no reason therefore to go over the same ground here. All that need be added here is to impress on the reader the absolute necessity that there should be lateral window light for every bed in the ward. In order to secure this it is necessary that each bed should be placed between two windows; the custom, so prevalent in Continental and American hospitals, of placing two beds alongside each other between two windows is highly undesirable.

The ward windows should be so arranged that two sides at least of the ward—the south-west and north-east sides—are window lit; by a suitable arrangement of the annexes it is also possible to secure window light on the south-east and north-west sides, though this is not absolutely necessary. Lighting on three sides is common enough in hospitals built on the pavilion system but not so common in wards of the block or corridor type.

With regard to window surface, no absolute rule can be laid down, as such proportion must be judged largely by the individual requirements of the institution. It is possible, however, to give a certain irreducible minimum. German architects put this at two square metres of window surface per bed for a ward with windows arranged only on one side, and a third of that surface for a ward which is lighted on two sides. Other constructors—for instance, Galton—take the cubic air space of the ward into consideration, which works out at approximately one square metre of window surface per bed. Estimating the amount of floor space required per patient at twelve square metres (Depage), the average minimum window-surface usually given in a modern hospital is two metres. From the table given in a former article it can easily be seen how few of the older hospitals even approach that minimum. It may be added that in Belgium a minimum of three and a half square metres per bed is required for the new hospitals; a minimum which is much more in consonance with modern hygienic requirements.

The size of the windows, in order to give this amount of window surface, must be large. It is the rule to carry the windows higher towards the ceiling and lower towards the ground nowadays than was formerly the case. Some architects advise that the window should be carried right up to the ceiling and down to the floor, but it is usually thought sufficient to stop within a foot of either. Depage and Vandervelde recommend that the windows be carried to within twenty-five centimetres of the ceiling, and brought down to seventy-five centimetres from the floor. The window panes should be of large size; nothing can be more unsuitable for a hospital ward than the so-called " artistic " window panes, in which a window consists of a dozen or more small panes in lead frames; such windows are difficult to clean and accumulate dust, while their artistic merits are problematical; utility and simplicity, and not the slavish following of garden city models, should be the aim of the architect in constructing a hospital.

It is very desirable that all hospitals in cold climates should be provided with double windows. These are of course more expensive than the ordinary single window, but they shut out not only cold but noise as well. Their main objection is that they shut out a certain amount of light. The distance between the two window panes should be relatively large. Whether single or double windows are used, the panes should be of clear, polished glass; ribbed, studded, bossed, or frosted glass is never to be used for the ward windows, although such glass is often very useful in certain of the annexes—for instance, in the bathroom and lavatories.

### Some Favourite Types.

Various types of windows are now used in hospitals, and most architects have a special predilection in favour of one or other variety. Perhaps the best type, and the one most generally suitable, is that which allows the window to be opened outwards in two portions, or even in one, and to be swung at an angle to the wall; this type of window facilitates ventilation to a very large extent, especially in summer. Swinging windows,

* Previous articles have appeared in The Hospital of Oct. 7, 14, 21, 28, Nov. 4, 18, Dec. 9, 30, and Feb. 10.

on the type of the Eyraut system, or the double window used at Dresden, have several advantages, but are not so simple as the ordinary laterally hinged types. Windows of the Ehrcke-Bley system are very convenient, but are dusty and difficult to keep clean: Stumpf's "Reform" type which is used in some hospitals is a modification of this system which presents certain advantages. Of the double-window type one of the best is that used in the Franz-Joseph hospital at Vienna, which has been described in detail by Ruppel in his "*Anlage und Bau von Krankenhäuser.*" The school windows designed by Cristoph and Unmack on the Döcker system appear to be very suitable for hospital use (Thel).

Necessary Details.

Frames with cords and pulleys, and complicated combinations such as are to be seen in some of the American clinics, are best avoided. The simpler and plainer the window and its frame, the better can they be kept clean and in order. It need scarcely be added that only the best materials should be used in their construction, and that they should be periodically inspected by a member of the hospital staff detailed for this duty. The window-sills should be as plain as possible where they are present. When the modern type of large-sized low window is used, they are usually superfluous. With the older higher type of window a window-sill is usually constructed; in that case it should be provided with a plain marble slab or enamelled paint surface. The window fastenings should be of a simple kind, and should be made to answer their object, not, as is often the case, merely to add to the worries of the ward sister.

Curtains, which are used at some of the German hospitals—notably at the Virchow—greatly add to the appearance of the ward, but should be dispensed with as they collect dust. Venetian blinds or pull-down blinds are also used but are open to the same objections; where shade is required it is generally preferable to secure it by employing screens round the bed. There is no need to be frightened at too much sun in a hospital.

The Ward Doors.

Equally important and worthy of consideration are the ward doors. These should be of firm, strong construction, and it must be held as a rule, which should never be departed from, that they must open outwards. There should be a door at each extremity of the ward. The doors should fit exactly in their frames; if there is the least bit of open space left between door and frame, currents of air are inevitably created. When the doors are made of inferior wood they are liable to shrink, and the adjustment between door and frame consequently becomes inexact; in that case the discrepancy may be remedied by rubber guards, but it is much better to see that the doors are of the best construction in the first place than to rely on such makeshifts later on.

Lightness and solidity are the main requirements in a hospital ward door. It ought to be so constructed that it can be opened and closed with the greatest ease, and its hinges, locks, handles, and connections ought to be of the best material. Double doors have no material advantages in a ward which is well ventilated and well constructed; they are often used in some of the annexes, especially in the isolation rooms. The best material to employ for the ward door is well-seasoned wood. Oak is most generally preferred; metal doors are unsuitable for the wards, but are generally used in the operating theatres.

Should the door be in one piece or with double wings? Both varieties are used, and have their advantages; perhaps the single door, in one solid piece, is better. Whichever form is used, the lower edge of the door should be flush with the ward floor; a door lintel is absolutely undesirable. The size of the door should be such that a bedstead or a transport waggon, with an attendant or nurse alongside, can easily pass between the door posts. A width of four feet between the posts is the minimum. Swinging doors opening both ways are sometimes used, but are apt to get out of order and present no distinct advantages over the door that opens only outwards. The addition of some simple apparatus to prevent slamming is desirable; it must be fixed on the outside of the door.

All doors should have a smooth, polished surface —as, for instance, is well seen in the Gilmour patent door, both sides of which have a smooth surface—which should be kept in perfect order by daily care and attention. It is better not to paint the surface; metallic doors, of course, should have a coat of enamel paint. The hinges should be kept well oiled, so that the door opens noiselessly. Locks are unnecessary on ward doors, and are better dispensed with; they should, however, be provided for the doors of all the annexes, especially for those of the

